# Natural Selection of a Virus-Protective FUT2 Variant Following the Transition to Agriculture

**DOI:** 10.1093/molbev/msaf243

**Published:** 2025-10-24

**Authors:** Johan Nordgren, Richard Ågren, David Ziliang Hu, Magdalena Neijd, Ainash Childebayeva, Kay Prüfer, Marie Hagbom, Lennart Svensson, Hugo Zeberg

**Affiliations:** Department of Biomedical and Clinical Sciences, Linköping University, Linköping SE 581 83, Sweden; Department of Physiology and Pharmacology, Karolinska Institutet, Stockholm SE 71 77, Sweden; Department of Physiology and Pharmacology, Karolinska Institutet, Stockholm SE 71 77, Sweden; Department of Biomedical and Clinical Sciences, Linköping University, Linköping SE 581 83, Sweden; Department of Archaeogenetics, Max Planck Institute for Evolutionary Anthropology, Leipzig DE 04103, Germany; Department of Anthropology, University of Texas at Austin, Austin, TX 78712, USA; Department of Archaeogenetics, Max Planck Institute for Evolutionary Anthropology, Leipzig DE 04103, Germany; Department of Biomedical and Clinical Sciences, Linköping University, Linköping SE 581 83, Sweden; Department of Biomedical and Clinical Sciences, Linköping University, Linköping SE 581 83, Sweden; Department of Medicine Solna, Karolinska Institutet, Stockholm SE 171 77, Sweden; Department of Physiology and Pharmacology, Karolinska Institutet, Stockholm SE 71 77, Sweden; Department of Evolutionary Genetics, Max Planck Institute for Evolutionary Anthropology, Leipzig DE 04103, Germany

**Keywords:** natural selection, viruses, protective, FUT2, variants, transition

## Abstract

Common enteric viruses rely on sugars mediated by the galactoside 2-alpha-L-fucosyltransferase 2 (FUT2) enzyme to infect host cells. Analyzing 4,343 ancient genomes, we map a premature stop codon in *FUT2*, which was introduced into Europe by migrating Anatolian farmers ∼6000 BC and provide evidence for positive selection. Using data from ∼700,000 present-day individuals, we confirm its protective effect against viral gastroenteritis. Experiments with intestinal organoids reveal that only homozygous carriers are protected against noroviruses and rotaviruses but not sapovirus. Our rotavirus findings resolve a long-standing contradiction between epidemiological data and experiments. Contrary to previous reports, we find no association between FUT2 loss of function and *Helicobacter pylori* infection. However, carriers exhibit an increased risk of gastric ulcers and gallbladder disease, associations replicated with an independent loss-of-function variant in East Asia. These findings suggest that the transition to agriculture and increased pathogen exposure drove positive selection of this allele.

## Introduction

Pathogens have influenced human evolution through local adaptations that confer resistance to endemic diseases, such as malaria resistance in Sub-Saharan Africa (Aidoo et al. 2002). As modern humans migrated, especially during the “Out-of-Africa” exodus 50,000 to 70,000 years ago, they encountered new pathogens, leading to genetic adaptations evident in contemporary genomes. These migrations also brought humans into contact with archaic species such as Neanderthals and Denisovans, each with their own evolutionary histories and pathogen interactions. Recent studies have shown that genetic variants inherited from these archaic humans influence immune responses to various infectious diseases (Zeberg and Pääbo 2020, 2021).

Beyond environmental changes from migration or climate shifts, human lifestyles can influence pathogen interactions (Bharti 2021). Arguably, the most substantial shift in lifestyle was the transition from hunter-gatherer societies to sedentary agriculture. This change impacted infectious diseases in two key ways: increased exposure to zoonotic diseases due to close contact with livestock and higher transmission rates resulting from reduced social distancing among humans. These factors introduced new immunological challenges that may have driven the selection of genetic variants conferring resistance to infections.

Loss-of-function alleles in the *FUT2* gene are examples of genetic variants that confer protection against infectious diseases. *FUT2* encodes the enzyme galactoside 2-alpha-L-fucosyltransferase 2, which synthesizes type I histo-blood group antigens on the mucosal surfaces of the gastrointestinal, respiratory, and genitourinary tracts, as well as in body fluids (Nordgren et al. 2016). These antigens are crucial in susceptibility to various pathogens (Cooling 2015). Epidemiological studies (Thorven et al. 2005; Rydell et al. 2011; Kambhampati et al. 2016; Nordgren et al. 2016; Sharma et al. 2020) have shown that individuals lacking functional FUT2 are less susceptible to rotavirus and norovirus, the leading causes of viral gastroenteritis worldwide (Bányai et al. 2018). However, while previous functional studies using intestinal organoids have demonstrated a FUT2 dependence on the infection of norovirus (Ettayebi et al. 2016), studies with laboratory-cultivated rotavirus in organoids or in other susceptible cell types have not (Saxena et al. 2015; Barbé et al. 2018), suggesting this discrepancy to epidemiological findings may be affected by cell culture adaptation.

The most common loss-of-function allele introduces a premature stop codon at position 143 in the encoded protein (chr19:48,703,417 G>A, *hg38*; rs601338). In this study, we investigate the evolutionary history of this allele in Europe using ancient DNA from 4,343 genomes spanning the past 10,000 years. We also provide a functional assessment of carriership by using human intestinal organoids to evaluate susceptibility to noroviruses, rotaviruses, and sapoviruses. Using a clinical rotavirus strain, we further resolve the discrepancy of FUT2 dependence between epidemiological observations and experimental studies using laboratory-cultivated rotaviruses.

## Results

### The Increase in Allele Frequency of the FUT2 Truncation Allele Follows the Transition to Agriculture

**Fig. 1.**
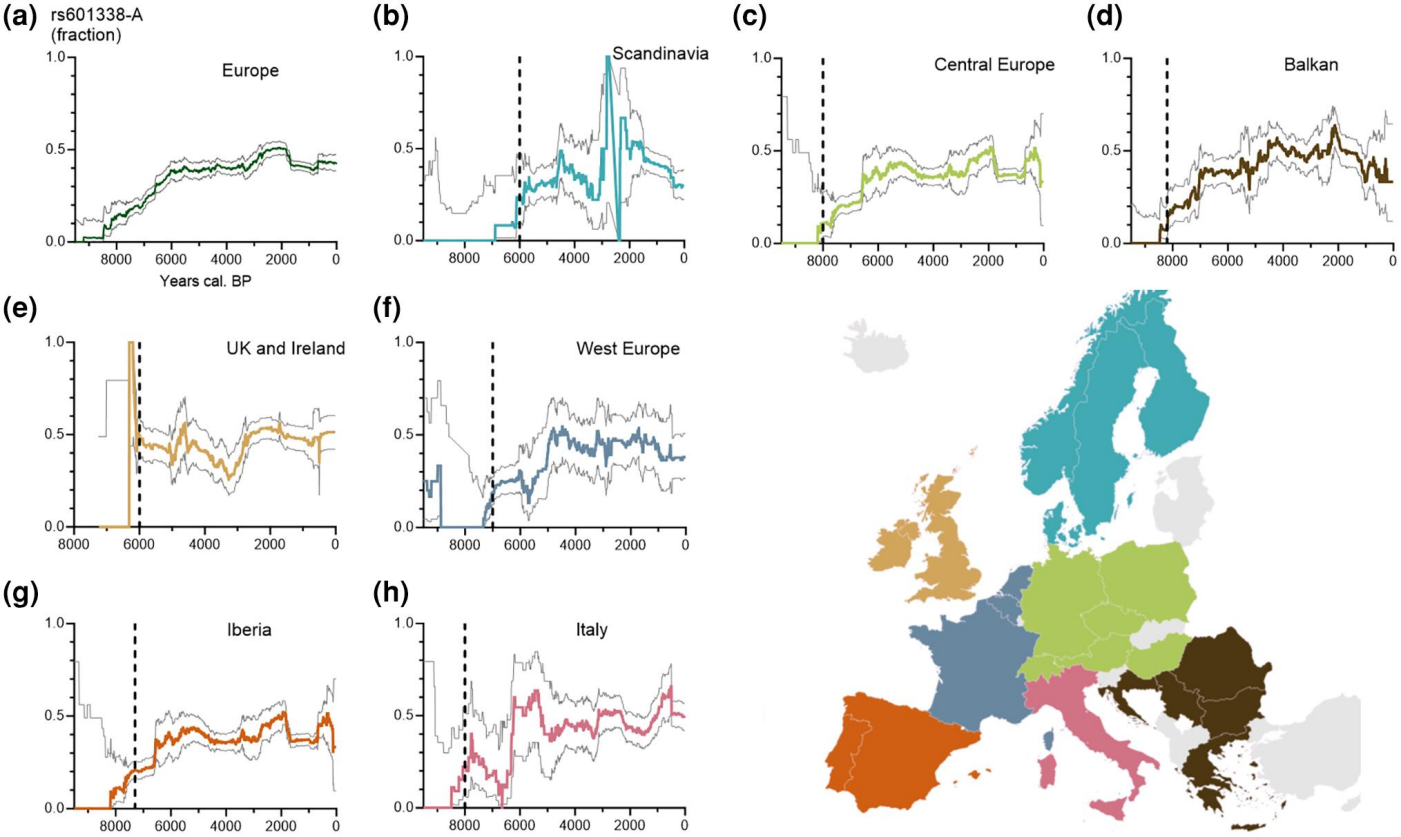
Evolutionary history of the enteritis-protective FUT2 truncation allele in relation to introduction of farming. Gray lines show 95% CIs. Large uncertainty for older times is due to small sample sizes. Vertical dashed line indicates when farming was introduced into the region (Shennan 2018). a) Frequency trajectory for the FUT2 truncation allele in Europe over the last 9,500 years. b to h) Frequency trajectories for the FUT2 truncation allele in Scandinavia b), Central Europe c), Balkan d), the United Kingdom and Ireland e), West Europe f), Iberia g), and Italy h). The dotted vertical bars represent suggested timepoints for introduction of farming. See Table S1 for countries included in the regions.

The truncation polymorphism in *FUT2* is included in the “1240k” capture array (Fu et al. 2015), which is utilized in over 70% of ancient human DNA studies (Rohland et al. 2022), facilitating its analysis in ancient genomes. Since most ancient DNA studies focus on European remains less than 10,000 years old, we focused on this period and region. From 7,599 ancient European genomes in the Allen Ancient DNA Resource (Mallick et al. 2024; Methods), we identified 4,343 genomes with genotype calls at the position of interest (chr19:48,703,417; *hg38*). To address periods with sparse data, we binned the data into 1,000-year epochs and plotted it in 10-year increments within these windows, achieving high-resolution trends while maintaining sufficient statistical power. We also expanded our dataset by including allele frequencies of two variants in high linkage disequilibrium (*r*² > 0.99, rs516246 and rs492602) with the FUT2 truncation allele for individuals lacking direct genotype calls. Our analysis revealed virtually no carriers of the FUT2 truncation allele before 8,000 years before present (BP). After this point, the allele frequency gradually increased, reaching 37% [95% confidence interval {CI}: 32 to 42%] by 6,000 years BP (Fig. 1a), similar to the present-day frequency of 44% observed in European populations (1000 Genomes Project; 451 alleles in 511 individuals).

**Fig. 2.**
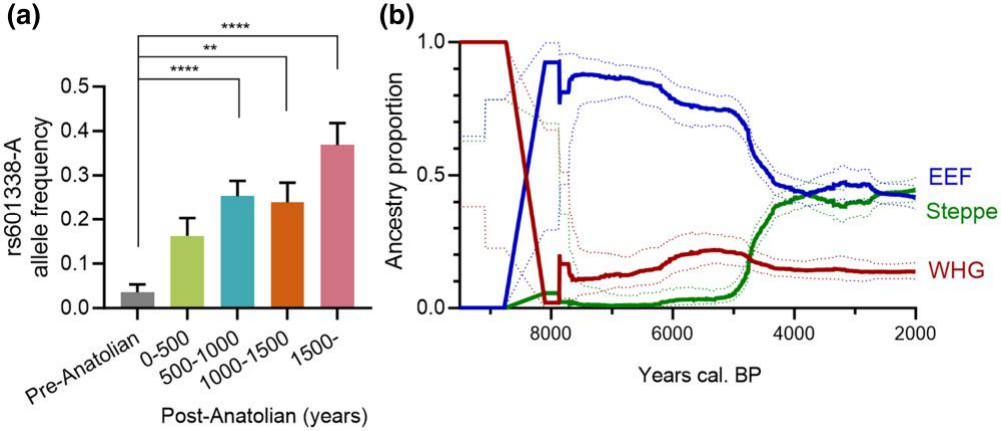
Increase in FUT2 truncation allele frequency following the introduction of agriculture. a) The FUT2 truncation allele frequency in Europe increased from 500 years after Anatolian entry. The studied European countries are listed in Table S1. b) Ancestry fractions in Europe, subgrouped by WHGs, EEFs, and Steppe populations, as shown. The rise in the FUT2 truncation allele frequency in Europe has taken place after Anatolian entry. Data are from Patterson et al. (2022). The samples used for this plot are listed in Table S2. Analysis of variance with multiple comparisons, *P* < 0.01, **; *P* < 0.0001, ****.

The period during which the FUT2 truncation allele increased in frequency coincided with the introduction of agriculture in Europe. Thus, to determine if this rise was associated with farming, we divided Europe into seven regions (Fig. 1b to h; Table S1 and Figure S1), each adopting agriculture at different historical times (Shennan 2018). Crucially, we possessed ancient DNA data from before the agricultural transition in these areas, enabling us to estimate allele frequencies before the onset of farming. We observed that following the introduction of agriculture, the allele frequency increased sharply, from virtually absent to 30% to 50% in Europe (Fig. 2a).

Present-day Europeans derive their genetic ancestry from three main sources: Western hunter-gatherers (WHGs), early European farmers (EEFs) (approximately equivalent to “Anatolians'), and steppe populations associated with the Yamnaya culture. EEFs arrived in Europe around 9,000 to 8,000 years BP, while steppe migrations occurred 6,000 to 5,000 years BP (Haak et al. 2015). Since the FUT2 truncation allele is virtually absent in samples older than 9,000 years BP, we infer that its allele frequency was close to zero among WHGs. To estimate its frequency among EEFs and steppe peoples, we analyzed a dataset of 1,229 ancient individuals (Patterson et al. 2022). For EEFs, we selected 61 individuals aged at least 6,000 years BP with over 90% EEF ancestry. Among the 30 with genotype data for the truncation allele, the allele frequency was 31% (95% CI: 18% to 51%). For steppe populations, we selected 59 individuals older than 4,000 years BP with more than 60% steppe ancestry. Among the 34 with genotype calls, the allele frequency was 35% (95% CI: 21% to 52%). Increasing the ancestry threshold or age criterion to identify steppe individuals would have significantly reduced the sample size. Using ancestry painting techniques (Materials and Methods), we also analyzed 99 present-day Finnish genomes (Auton et al. 2015), using Sardinians as a proxy for EEF ancestry (Haak et al. 2015; Chiang et al. 2018) and Kalash for steppe ancestry (Rahman et al. 2020). The estimated allele frequencies were 31% (95% CI: 24% to 37%) for the EEF component and 13% (95% CI: 2% to 47%) for the steppe component, consistent with our findings from the ancient samples.

### Selection on the FUT2 Truncation Allele

To determine whether natural selection acted on the FUT2 truncation allele, we first assessed if genetic drift in a stable, panmictic population could explain the observed increase in allele frequency from approximately 2% to 50% over 300 generations (∼8,700 years, assuming a 29-year generation time; Langergraber et al. 2019). Although the Fisher–Wright model lacks a closed-form solution for allele frequency trajectories, we can calculate the variance. Using standard equations (Materials and Methods) and assuming an effective population size of 10,000 individuals, we computed the standard deviation of allele frequency change under drift to be about 2.4%. This small variance indicates that genetic drift alone cannot account for the substantial increase.

Rapid changes in allele frequencies are often due to shifts in population ancestry from migration and admixture rather than selection. To illustrate the ancestry shifts in Europe during the Holocene, we extracted all samples dated between 10,000 and 1,500 BP from Fig. 1a for which ancestry fractions were available from a previous analysis (Patterson et al. 2022). The results are shown in Fig. 2b. As can be seen, Europe underwent a significant demographic turnover around 8,500 BP with the arrival of EEFs, who largely replaced the indigenous WHGs. We assumed the FUT2 truncation allele was introduced into Europe by these farmers at a frequency of 31% (based on the genotypes from the 30 EEFs described above). Despite a slight decrease in EEF ancestry between 8,500 and 5,000 BP (Fig. 2b), the allele frequency continued to rise during this period.

To test for selection, we evaluated the probability that the actual allele frequency increase from 31% (i.e. the allele frequency at 8,500 BP) to 39% (i.e. the allele frequency at 5,600 BP) over 100 generations occurred due to genetic drift alone. By comparing with allele frequency distributions in the absence of selection (using a fitted beta distribution, see Materials and Methods), we found that the observed increase in allele frequency is unlikely under drift alone (*P* = 8.9 × 10⁻³). This suggests that positive selection contributed to the allele's rise in Europe.

Between 5,000 and 4,000 BP, EEF ancestry decreased due to steppe migrations (Fig. 2b), yet the allele frequency of the FUT2 truncation allele remained constant. Given that the allele frequency before the arrival of steppe people was approximately 40%, and the frequency among steppe individuals was about 35%, we would not expect a major change in allele frequency due to this population turnover.

### Homozygous Carriers of the FUT2 Truncation Allele Are More Protected Against Viral Gastroenteritis

**Fig. 3.**
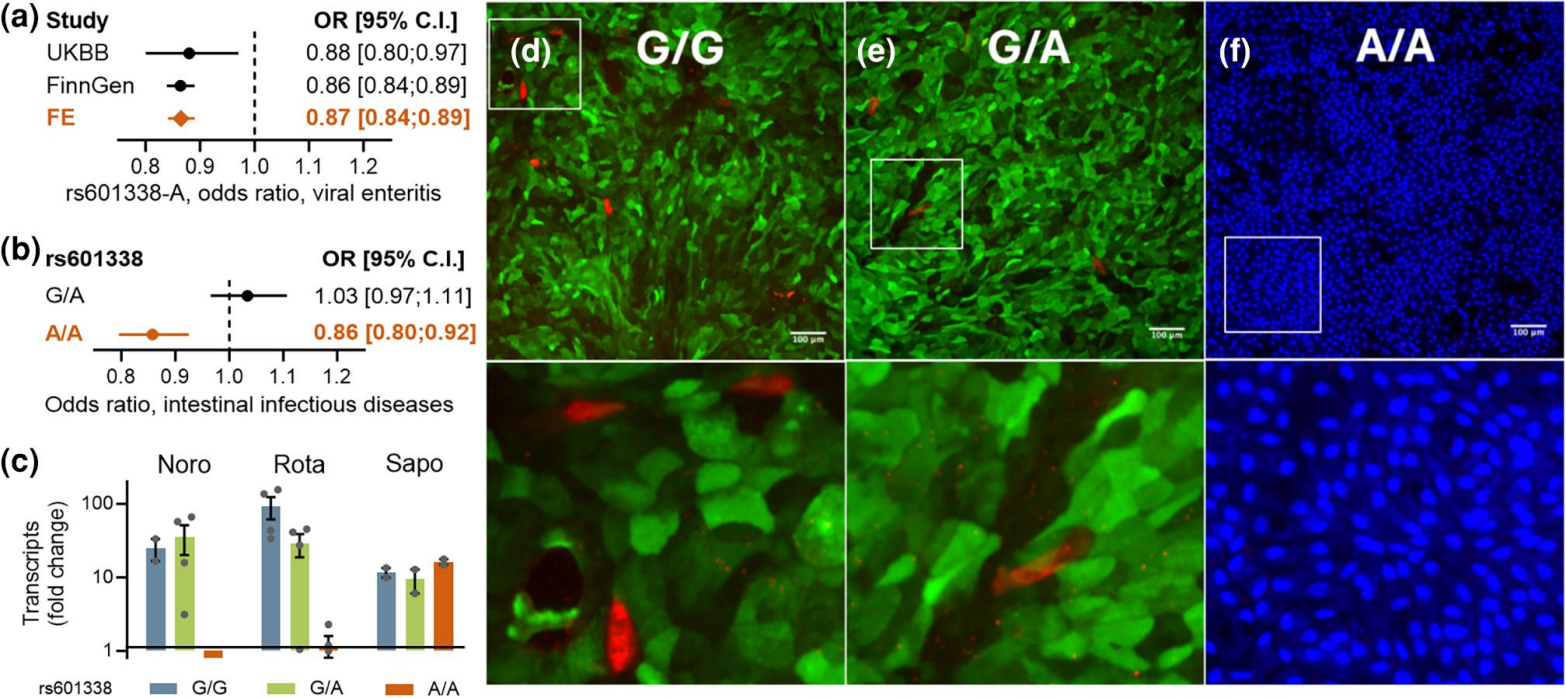
Homozygous rs601338-A carriership is associated with protection against viral gastroenteritis. a) Meta-analysis of the association between rs601338-A and the phenotype “viral gastroenteritis” in the UK Biobank and FinnGen (freeze 11) studies (Heyne et al. 2023). b) In FinnGen, the association between rs601338-A and reduced risk for viral gastroenteritis is solely present in homozygous carriers. c) Norovirus, rotavirus, and sapovirus replication in enteroids of different *FUT2* genotypes. rs601338-A/A carriership did not affect the susceptibility for sapovirus. The bars depict a fold increase of viral RNA after 72 h compared to 2 h, as measured by qPCR. d to f) Immunofluorescent staining of rotavirus (red) and α1,2-fucose (green) of human enteroids carrying rs601338-G/G d), G/A e), and A/A f) genotypes. Lower row depicts high-magnification images (corresponding to the white boxes above). α1,2-Fucose is absent in intestinal organoids that are homozygous for the FUT2 truncation allele. Nuclei are stained in blue (DAPI). The scale bar equals 100 µm.

Loss-of-function mutations in *FUT2* are known to confer protection against common causes of viral gastroenteritis (Thorven et al. 2005; Rydell et al. 2011; Kambhampati et al. 2016; Nordgren et al. 2016; Sharma et al. 2020). Using the FinnGen biobank (freeze 11), we found that the truncation allele is associated with a reduced risk of “viral and other specified intestinal infections” [International Classification of Diseases {ICD}-10 A08], with an odds ratio (OR) of 0.86 per allele (95% CI: 0.84 to 0.89, *P* = 8.8 × 10⁻²²; Fig. 3a). This makes *FUT2* the second most significant locus after the human leukocyte antigen (HLA) region for this outcome. The association was replicated in the UK Biobank with a similar effect size (OR = 0.88, 95% CI: 0.80 to 0.97, *P* = 8.2 × 10⁻³; Fig. 3a). However, these clinical outcomes do not differentiate among virus types or clarify whether the protective effect is specific to homozygous carriers.

Previous research suggests that only homozygous loss of function of FUT2 (the “nonsecretor” phenotype) confers protection against viral gastroenteritis, while heterozygous carriers do not (Thorven et al. 2005; Kambhampati et al. 2016). To investigate this, we analyzed FinnGen data comparing recessive and additive genetic models (Heyne et al. 2023). Although data specific to viral intestinal infections were unavailable, we found that for the broader category of “intestinal infectious diseases” (ICD-10 A00–A09), protection was observed only in homozygous carriers (Fig. 3b).

To provide functional evidence supporting the recessive protective effect and its specificity to certain viruses, we conducted experiments using human intestinal enteroids. These organoids originated from stem cells of jejunum biopsies of one homozygous wild-type individual, one heterozygous carrier, and one homozygous truncation allele carrier. The enteroids were infected with a clinical sample of rotavirus, norovirus, and sapovirus, respectively. Viral replication assessed by quantitative polymerase chain reactions (qPCR), complemented with immunostaining for rotavirus, revealed that organoids from homozygous wild-type and heterozygous individuals had similar susceptibility to rotavirus and norovirus. In contrast, organoids from homozygous truncation allele carriers showed markedly reduced susceptibility to rotavirus and no replication of norovirus (Fig. 3c). Using a laboratory-cultivated rotavirus strain (F45), we observed similar replication in all enteroids (data not shown), aligning with previous reports of FUT2 independence for laboratory-cultivated rotavirus strains (Saxena et al. 2015; Barbé et al. 2018). All enteroids further supported sapovirus replication (Fig. 3c), in agreement with previous epidemiological and functional evidence that sapovirus infection is independent of FUT2 function (Bucardo et al. 2012, Euller-Nicolas et al. 2023; Matussek et al. 2015). We confirmed secretor status using lectin staining, noting that nonsecretor organoids (homozygous for the truncation allele) do not express α1,2-fucose (Fig. 3d to f).

### Cost of Carriership of the FUT2 Truncation Allele

**Fig. 4.**
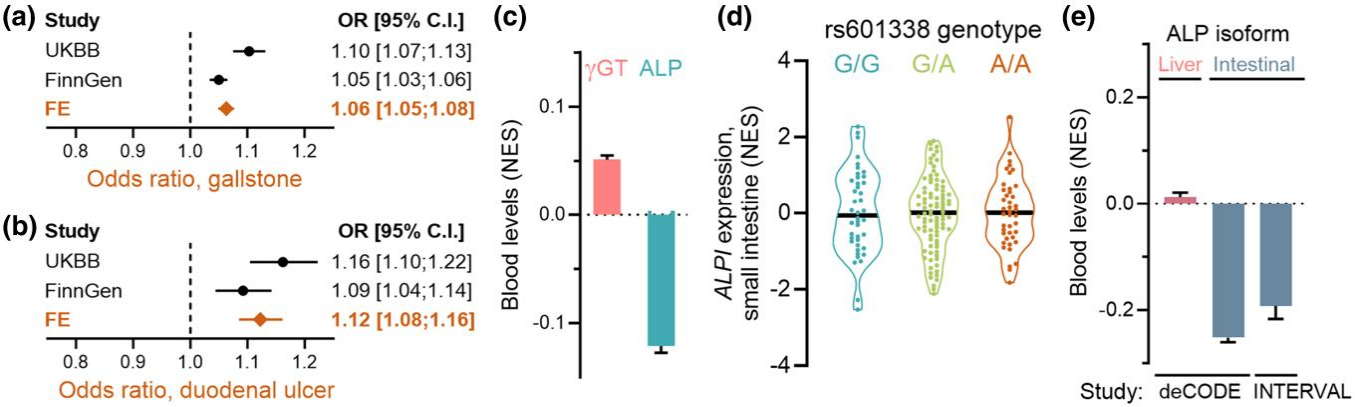
The FUT2 truncation allele is associated with gastrointestinal conditions and altered blood enzymes. a and b) Associations between rs601338-A carriership and gallstones (a) and duodenal ulcer b) in the UK Biobank and FinnGen (freeze 11) studies. Fixed-effects meta-analysis (FE) is performed using both biobanks. c) Association between rs601338-A carriership and blood γ-glutamyl transferase (γGT) activity and ALP activity. The data are derived from the UK Biobank (Klimentidis et al. 2020; Barton et al. 2021). d) Expression of *ALPI* (encoding intestinal ALP) in the small intestine. Data from 174 individuals in the GTEx database (GTEx Consortium 2013). e) Blood levels of liver and intestinal ALPs. The rs601338 genotype does not impact the levels of liver ALP (pink). Proteome data are from the deCODE (Ferkinstad et al. 2021) and INTERVAL studies (Sun et al. 2018). NES, normalized effect size.

We next investigated potential detrimental consequences of carriership. A phenome-wide association study in FinnGen (up to 453,733 individuals; 2,445 phenotypes) identified 21 significant associations (*P* < 2.04 × 10⁻⁵; Table S3), grouping into five traits beyond viral intestinal infections. Only gallstones and acute laryngitis and tracheitis replicated in the UK Biobank (500,000 individuals; 1,272 phenotypes). Additionally, using the UK Biobank as the discovery set and requiring similar effects in FinnGen, we identified duodenal ulcers as significantly associated. Meta-analysis of both biobanks confirmed that the FUT2 truncation allele is associated with an increased risk of gallstones, duodenal ulcers (Fig. 4a and b), and acute laryngitis and tracheitis (Figure S2).

To further investigate these associations, we analyzed laboratory blood data from the UK Biobank. The FUT2 truncation allele was significantly associated with elevated gamma-glutamyl transferase (γGT; *P* = 3.0 × 10⁻¹⁵⁵; Fig. 4c), a marker associated with liver disease and bile duct obstruction, and decreased alkaline phosphatase (ALP) activity (*P* = 1.2 × 10⁻^723^; Fig. 4c), consistent with previous reports in secretor-negative individuals (Domar 1992). RNA expression analysis in the Genotype-Tissue Expression database (Lonsdale et al. 2013) showed no effect on genes encoding two ALP isoenzymes, the “liver” (encoded by *ALPL*) and “intestinal” (encoded by *ALPI*; Fig. 4d). The names of these enzymes relate to where they were first discovered, although these enzymes are not exclusively expressed in the liver and intestine, respectively. Proteomics data revealed markedly lower levels of circulating intestinal ALP in carriers of the truncation allele (Fig. 4e), explaining the reduced ALP activity and aligning with earlier findings. The strongest genome-wide associations for intestinal ALP across two European biobanks were at the *FUT2* (*P* = 2.4 × 10⁻^217^) and *ABO* loci (*P* = 2.0 × 10⁻^256^).

Given that a primary cause of gastric and duodenal ulcers is *Helicobacter pylori* infection, we investigated if the FUT2 truncation allele is associated with *H. pylori* serology from 4,304 Britons. (Backman et al. 2021), with a seropositivity of 35% (UK Biobank data field 23073, Bycroft et al. 2018). We find that *H. pylori* serology status is not associated with secretor status (OR = 1.00; 95% CI: 0.90 to 1.10; *P* = 0.94).

### An East Asian FUT2 Missense Variant Replicates the Phenotypic Associations

**Fig. 5.**
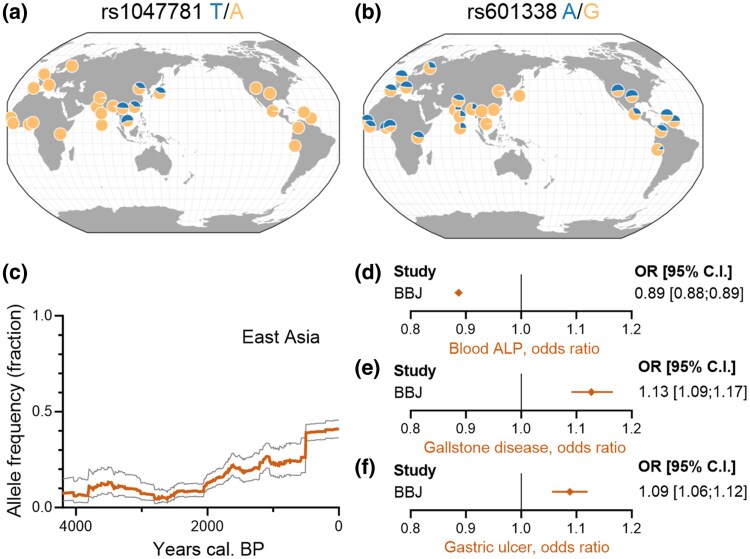
The East Asian-specific norovirus-protective FUT2 missense variant rs1047781-T replicates the gastric phenotypes. a and b) Present-day geographic distribution of the gastroenteritis-protective variants rs1047781-T a) and rs601338-A b). c) Allele frequency of the rs1047781-T variant in East Asia in the Allen Ancient DNA Resource. d to f) rs1047781-T is associated with reduced blood ALP levels d) and increased risk for gallstone disease e) and gastric ulcer f) in Biobank Japan (Sakaue et al. 2021).

Despite extensive interactions between Eurasian populations over the past 2,400 years (Qin et al. 2015) and the early advent of Neolithic farming in East Asia dating back 7,700 years (Zong et al. 2007), the FUT2 truncation allele (rs601338-A) remains absent in East Asian populations. This absence suggests the existence of alternative protective mechanisms against infectious enteritis in East Asia, which may have precluded positive selection for this allele in these populations. Epidemiological studies have identified a FUT2 missense variant (rs1047781-T, encoding I129F) that protects against rotavirus and norovirus gastroenteritis (Ferrer-Admetlla et al. 2009). Unlike the truncation allele, this missense variant is highly specific to East Asia (Fig. 5a and b). Analyzing the allele frequency of this variant over time in East Asian genomes, we observed that it began to rise significantly around 2,000 years BP, approximately 6,000 years after the introduction of farming in the region (Fig. 5c). In modern populations, its frequency has increased to about 40%.

To investigate whether the East Asian missense variant is associated with similar phenotypic effects as the truncation allele, we analyzed phenotypic associations using data from Biobank Japan (Sakaue et al. 2021). Our analysis revealed that the missense variant is associated with reduced blood ALP levels (Fig. 5d) and increased risks of gallstones (Fig. 5e) and duodenal ulcers (Fig. 5f), mirroring the associations observed for the truncation variant. These findings illustrate that two distinct and independent FUT2 mutations can exhibit similar phenotypic effects. While FUT2 reduction-of-function mutations confer protection against viral gastroenteritis, they may also increase the risk of other associated diseases.

## Discussion

In this study, we traced the history of a FUT2 truncation allele in Europe throughout the Holocene. Prior to the advent of agriculture, this allele was virtually absent, suggesting that it likely arrived with populations migrating from Anatolia. Building on earlier findings that homozygous carriers of inactivating FUT2 mutations gain protection against gastroenteritis, we propose that the increased prevalence of enteric viruses, spurred by denser, sedentary Neolithic communities, drove positive selection for the truncated FUT2 allele. Indeed, we find evidence of such selection using ancient European genomes in the time window between 8,500 and 5,000 BP. These results align with the long-standing proposition (e.g. Barrett et al. 1998) that the Neolithic transition, marked by farming and higher pathogen loads, fundamentally reshaped human susceptibility to infectious diseases.

Across six of the seven regions examined, the FUT2 truncation allele frequency gradually rose over time following the introduction of agriculture. However, the “UK and Ireland” region presents an exception, with a rapid increase in allele frequency (nearly 0% to about 50%) around 6,000 BP. This abrupt shift might reflect a population turnover. Subsequently, the allele frequency in this region declined from around 50% to 20% at approximately 3,200 BP, only to rebound again to 50%. These fluctuations may reflect broader demographic and cultural transformations, including the Late Bronze Age collapse (∼3,000 BP) and associated migrations from mainland Europe (Patterson et al. 2022).

Previous studies have suggested that inactivating mutations in FUT2 may confer protection against *H. pylori* infection (Ikehara et al. 2001; Magalhães et al. 2009). This observation appears at odds with our findings indicating an increased risk of duodenal ulcers in individuals with these mutations. However, Ikehara et al. (2001) relied on a smaller dataset (60 gastric biopsies), whereas our analysis of 4,304 Britons yielded no association between FUT2 truncation and *H. pylori* seropositivity. Additionally, we observed no protection against clinically diagnosed *H. pylori* infection; in fact, there was a suggestive trend in the opposite direction (ICD-10 B98: OR = 1.07; 95% CI: 0.99 to 1.16; *P* = 0.08; Backman et al. 2021). This trend might be attributed to an increased propensity of seeking medical attention for gastrointestinal symptoms in individuals carrying the FUT2 truncation allele. Such behavior could increase the likelihood of detecting *H. pylori* infection, thereby contributing to the observed trend. Another possibility for the increased risk of duodenal ulcers is that the fucosylation caused by FUT2 contributes to mucosal integrity.

Regarding cost of carriership of the FUT2 truncation allele, we note several strong phenotypic effects (Fig. 4a and b and Figure S2 and Table S3). However, whether these associations explain the present allele frequencies remain unknown, and we note that past diseases with high selective pressure on this allele may not be reflected in present biobanks. We observe a strong association between a functional FUT2 and higher intestinal ALP levels, in line with a previous report (Domar 1992). It has long been noted that intestinal ulceration is associated with an increase in intestinal ALP (Fodden 1953). However, at least to us, the causal relationship between ulcers, intestinal ALP, and FUT2 function remains obscure.

Building on previous experimental study design and findings (Saxena et al. 2015; Ettayebi et al. 2016; Euller-Nicolas et al. 2023), we performed a functional assay using human enteroids. We used organoids being homozygous for a functional FUT2 enzyme, homozygous for the FUT2 truncation allele, or heterozygous with one functional allele and one truncation allele. Infections with clinical samples of norovirus (GII.4), rotavirus (G8P[8]), and sapovirus (GI.1) supported epidemiological data; homozygous FUT2 truncation carriers were protected against norovirus and rotavirus but remained equally susceptible to sapovirus. In contrast, when we used a laboratory-cultured rotavirus strain (F45) of the same P[8] genotype, FUT2 function did not influence infectivity. These findings, in agreement with the previous work (Saxena et al. 2015; Barbé et al. 2018), underscore the importance of using clinical samples to understand FUT2-dependent susceptibility rather than relying solely on laboratory-adapted strains (Peña-Gil 2023).

We replicated associations for an independent missense FUT2 reduction-of-function variant in East Asia. The East Asian missense variant increases in frequency similarly to the FUT2 truncation allele in Europe (Fig. 5c), reaching comparable levels and suggesting similar selection pressures and plateauing frequencies. To date, we have limited evidence on major population changes from 6,000 years ago to the present (Wang et al. 2021a , b) in East Asia. Therefore, the situation is different compared to the several waves of migrations into Europe. However, one hypothesis would be that the initiation of the Silk Road around 100 BC (Peters 2019) may have introduced diseases such as certain variants of viral gastroenteritis.

Overall, our study highlights how the transition to agriculture and the resulting changes in human lifestyle and population density likely led to adaptive evolutionary changes. However, the positive selection of the FUT2 truncation allele, providing protection against viral gastroenteritis, also brought with it increased susceptibility to other conditions. The evolutionary dynamics of the FUT2 gene exemplify how human adaptation to new environmental pressures has shaped genetic diversity.

## Materials and Methods

### Ancient Genomes

For ancient genetic data, we used the Allen Ancient DNA Resource v.62. We analyzed European, West Asian, and East Asian genomes characterized using either the “1240k” capture array, which contains 1,233,013 positions (Fu et al. 2015), or shotgun sequencing. For analyzing the FUT2 truncation variant, we included genomes typed for rs601338 or its proxy variants rs492602 (chr19:48,703,160A>G, *hg38*) and rs516246 (chr19:48,702,915C>T, *hg38*), which are in high linkage disequilibrium (*r*² > 0.99 in Europeans from the 1000 Genomes Project). For East Asian genomes, only those containing rs1047781 (chr19:48,703,374A>T, *hg38*), which encodes the FUT2 missense variant, were included. It should be noted that the majority of the analyzed ancient genomes are pseudohaploid, a common feature due to the low coverage of ancient DNA sequencing.

### Ancient Allele Frequencies, Ancestry Proportions, and Selection

Allele frequency data spanning 0 to 10,000 calibrated years BP were smoothed using a rolling window of 1,000 years. For the subsequent analyses, data points were evaluated at 10-year intervals, and 95% binomial proportion CIs were calculated for each interval. Repeated statistical comparisons across time periods were performed using one-way analysis of variance, followed by Dunnett's multiple comparison correction to control for type I error. To estimate the variability in allele frequencies over time, we employed an approximation of a standard formula (Tataru et al. 2016) to calculate the variance of the distribution of allele frequencies: Var(*f*_1_) = *r* × *f*_0_ (1−*f*_0_)/(2 × Ne), where *r* is the number of generations, *f*_0_ is the initial frequency, *f*_1_ is the final frequency, and Ne is the effective population size. As per the Fisher–Wright model, the expected value of *f*_1_ was set to the initial frequency *f*_0_.

To formally test for selection, we modeled the distribution of allele frequency changes using a Beta distribution parameterized according to the mean (initial frequency *f*_0_) and variance derived from the Fisher–Wright model, as outlined above. The *P*-values were calculated directly from this Beta distribution. Although an approximation, this method provides a superior fit to our data compared to a normal distribution. To minimize confounding by temporal fluctuations in ancestry composition, our analyses were restricted to intervals characterized by stable ancestry proportions. This approach ensured that detected allele frequency changes were less likely to reflect external demographic shifts.

To characterize the ancestry shifts in Europe during the Holocene, we extracted all ancient DNA samples dated between 10,000 and 1,500 BP from the dataset shown in Fig. 1a (samples listed in Table S2). For inclusion, samples were required to have ancestry fraction estimates available from a previous analysis (Patterson et al. 2022). Ancestry proportions were visualized to illustrate demographic turnover events, with a particular focus on the replacement of indigenous WHGs by EEFs around 8,500 BP.

### Ancestry Painting

RFMix (Maples et al. 2013) was utilized to assign Anatolian and steppe haplotypes onto chromosome 19 for carriers of the FUT2 truncation variant. A reference panel was constructed using phased genotype data from available genomes from individuals with known Anatolian and steppe ancestries. For this, Human Genome Diversity Project data on 27 Sardinians, and for steppe, we used 23 Kalash from Pakistan as proxies. To evaluate ancestry contributions to the modern haplotype carrying the FUT2 truncation variant, 99 Finnish carriers were selected from the 1000 Genomes Project (Auton et al. 2015). Chromosome 19 was filtered using the strict callability mask from the 1000 Genomes Project (Auton et al. 2015) and a minor allele frequency cutoff > 5%. Next, the RFMix algorithm was used to estimate Anatolian (Sardinian) and steppe (Kalash) ancestry across the chromosomes, using standard parameters for a two-population model.

### Biobank Analyses

Phenome-wide associations were analyzed for the FUT2 truncation variant using the two European biobank studies: UK Biobank (Bycroft et al. 2018) and FinnGen freeze 11 (Kurki et al. 2023). The East Asian FUT2 missense variant associations were investigated using Biobank Japan (Sakaue et al. 2021). Gene variant dependence on expression was studied in plasma and tissue. Plasma protein quantitative trait loci data were retrieved from the Icelandic deCODE cohort (Ferkinstad et al. 2021) and the European INTERVAL cohort (Sun et al. 2018). The Genotype-Tissue Expression database (Lonsdale et al. 2013) was used to study the effects of FUT2 truncation on RNA expression.

In the case of single-variant associations, we directly extracted *P*-values from the genome-wide association study (GWAS) summary statistics provided by the respective biobanks. ORs were calculated by exponentiating the reported β-values (OR = exp(β)), as β represents the log-transformed OR. The 95% CIs were first computed on the β-scale by adding and subtracting 1.96 times the standard error (SE) and subsequently transformed to the OR scale. For meta-analysis of results from multiple biobanks, we applied an inverse-variance weighted method, as implemented in the R package metagen. To avoid floating point errors for *P* < 10⁻^300^, numerical approximation was performed based on Z-values using NIntegrate in Mathematica 12 (Wolfram Research, Inc., IL, USA).

### Viral Infection of Enteroids

Human intestinal enteroids were previously established from adult stem cells in jejunal biopsies from patients that underwent gastric bypass surgeries. The procedure has been reviewed and approved by the Swedish Ethical Review Authority (N00600/19). Enteroids carrying the wild-type, heterozygous, and homozygous FUT2 truncation alleles were cultured in IntestiCult Organoid Growth Medium (Stemcell Technologies, Vancouver, Canada), similarly as described previously (Ettayebi et al. 2016). For infection studies, human intestinal enteroids were differentiated into monolayers (Ettayebi et al. 2016). Single-cell suspensions were seeded in collagen-coated 96-well plates and after monolayer formation (day 2), the medium was replaced with Intesticult Organoid Differentiation Medium (STEMCELL Technologies, Vancouver, Canada). After 5 days of differentiation, monolayers were washed twice with advanced Dulbecco's Modified Eagle Medium (DMEM)/Nutrient Mixture F-12 (F12; Sigma, MO, USA) supplemented with gentamicin, 4-(2-hydroxyethyl)-1-piperazineethanesulfonic acid (HEPES), and GlutaMAX. Clinical viral samples were retrieved from routine clinical diagnostics, in line with guidance from the Swedish Ethical Review Authority (N00605/21). To every well of cells, a 1:100 dilution of clinical feces samples, positive for a human G8P[8] rotavirus, a GII.4 norovirus, or GI.1 sapovirus, was added. For norovirus and sapovirus, 500 μM of glycochenodeoxycholic acid (Sigma, MO, USA) was added to the fecal dilution. For infections with the laboratory-cultivated human rotavirus G9P[8] (strain F45), 1.5 × 10^2^ infectious particles/well were used. All samples were diluted in serum-free DMEM. Two hours postinfection, the monolayers were washed twice and fresh IntestiCult Organoid Differentiation Medium was added to the cells.

### Immunofluorescence Staining

At 24 h postinfection, human intestinal enteroids were fixed with 4% formaldehyde for 2 h. After washing with phosphate-buffered saline (PBS), cells were permeabilized with 0.3% Triton X-100 and treated with 3% bovine serum albumin (BSA) in PBS for 1 h at 37 °C. Following one PBS wash, cells were incubated with primary antibody (guinea pig-derived anti-rotavirus VP6, prepared in-house) and incubated for 90 min at 37 °C followed by four washes and secondary antibody addition (goat anti-guinea pig Alexa594; Jackson ImmunoResearch, PA, USA; #106-585-003) containing 4´,6´-diamidino-2-phenylindole (DAPI) for nuclear staining, for 1 h at room temperature. For α1,2-fucose staining, monolayers were incubated with fluorescein isothiocyanate-labeled lectin from *Ulex europaeus* (Sigma; #L9006). After four washes, 150 μL of PBS was added to each well before microscopy.

### Microscopy

Wide-field micrographs were acquired using the DMi8 microscope (Leica, Wetzlar, Germany). Postacquisition brightness/contrast adjustments were performed uniformly on all images using ImageJ (Schneider et al. 2012).

### Viral RNA Quantification

Viral RNA was extracted using the QIAamp Viral RNA Mini Kit (QIAGEN, Hilden, Germany). Following 2 and 72 h after viral infection, human intestinal enteroids were treated with AVL lysis buffer (QIAGEN) followed by RNA extraction that was subsequently stored at −80 °C until cDNA synthesis. cDNA synthesis was performed using the High-Capacity cDNA Reverse Transcription Kit (Applied Biosystems, MA, USA). For rotavirus (dsRNA), an initial denaturation was performed at 97 °C for 5 min before cDNA synthesis. The cDNA was subsequently used to quantify virus genes using qPCR. The qPCRs for norovirus, rotavirus, and sapovirus were performed using iTaq Universal Probes Supermix (Bio-Rad, CA, USA) on a CFX96 instrument (Bio-Rad) with primers and probes targeting the *ORF1–ORF2* junction for norovirus (Hagbom et al. 2021), the *ORF1* RNA polymerase-capsid junction for sapovirus (Oka et al. 2006; Varela et al. 2019), and the *NSP3* gene for rotavirus (Freeman et al. 2008; Bucardo et al. 2019).

## Supplementary Material

msaf243_Supplementary_Data

## Data Availability

Data generated or analyzed in this study are included in this article or publicly available. Script to produce Fig. 1 is available at https://github.com/DavidHu-Ki/FUT2_paper/.
