# Supplementary material for: Natural Selection of a Virus-Protective FUT2 Variant Following the Transition to Agriculture
Source: Mol Biol Evol. 2025 Oct 24;42(10):msaf243. doi: 10.1093/molbev/msaf243 (PMC12550361; doi:10.1093/molbev/msaf243)
Supplement: msaf243_Supplementary_Data [file msaf243_supplementary_data.pdf]

Supplementary materials for

Natural selection of a virus-protective FUT2 variant following the transition to agriculture

Johan Nordgren[1,*], Richard Ågren[2,*], David Hu Ziliang[2], Magdalena Neijd[1], Ainash Childebayeva[3], Kay Prüfer[3], Marie Hagbom[1], Lennart Svensson[1,4] and Hugo Zeberg[2,5,#]

[1] Department of Biomedical and Clinical Sciences, Linköping University
[2] Department of Physiology and Pharmacology, Karolinska Institutet
[3] Department of Archaeogenetics, Max Planck Institute for Evolutionary Anthropology
[4] Department of Medicine Solna, Karolinska Institutet, Stockholm
[5] Department of Evolutionary Genetics, Max Planck Institute for Evolutionary Anthropology

**Shared first authorship*
#Corresponding author; hugo.zeberg@ki.se

Supplementary figures

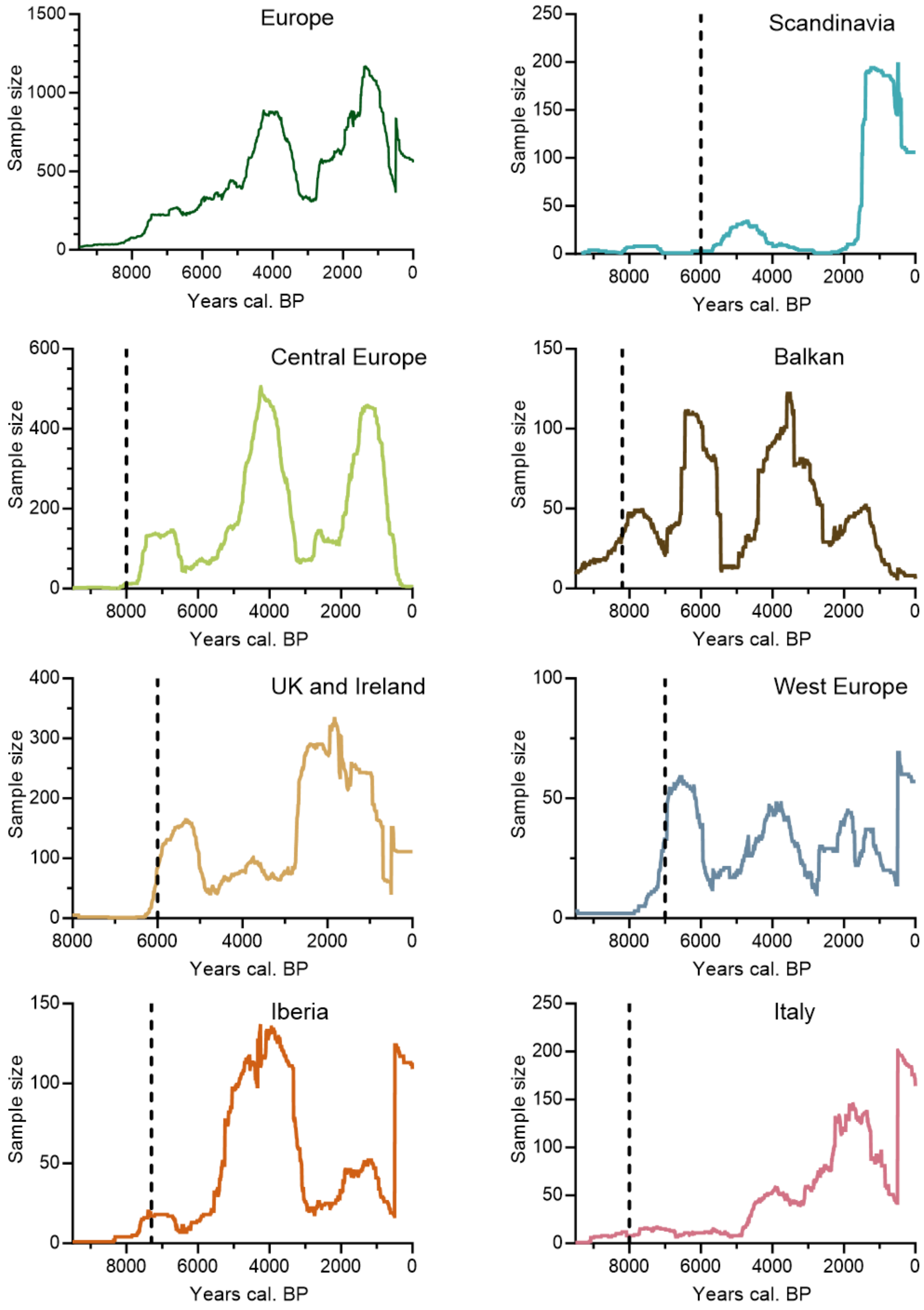

Supplementary Figure 1 | Sample sizes of individuals in FUT2 truncation allele frequency calculations. The regions correspond to Figure 1. The dotted vertical bars represent suggested timepoints for the introduction of farming. See **Supplementary Table 1** for countries included in the regions.

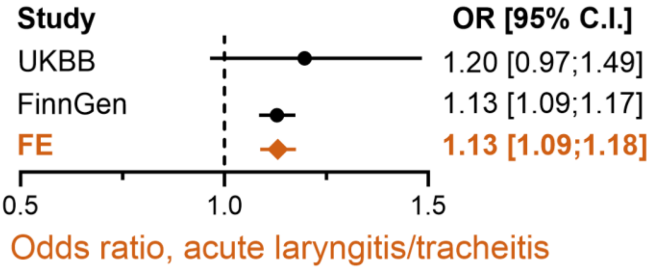

Supplementary Figure 2 | The FUT2 truncation allele is associated with airway inflammation. Association between rs601338-A carriership and acute laryngitis and tracheitis in the UK Biobank and FinnGen (freeze 11) studies. $p = 5.3 \times 10^{-10}$. Fixed-effects meta-analysis (FE) is performed using both biobanks.

Supplementary tables

Supplementary table 1 | European countries used in subregional carriership analyses.

**Region**	**Country**
Scandinavia	Norway
	Sweden
	Finland
	Denmark
Central Europe	Austria
	Czechia
	Germany
	Hungary
	Switzerland
	Poland
Balkan	Bulgaria
	Croatia
	Greece
	Romania
	Serbia
UK and Ireland	UK
	Ireland
Western Europe	France
	Belgium
	Netherlands
Iberia	Portugal
	Spain
Italy	Italy

Supplementary table 2 | Samples used in the ancestry fraction analysis in Figure 2B.

I6754	I5370	I8197	I6472	I13469	I7949	VIL004	I14801
I3025	I15944	I8198	I6475	I13471	I7629	I12410	I25508
I1880	I15947	I8199	I6588	I16109	I20517	I8340	I18838
I4918	I2796	I7587	I6587	I15644	I20516	I8212	I18834
I3537	I13898	I1970	I6584	I13468	I15955	I8341	I18832
PEN003	I2634	Aes25	I6539	I7959	I15961	I8214	I18837
PEN001_real1	I12314	I1272	I2419	I7962	I13787	I8210	I18833
PEN001_real2	I6677	I8364	I1388	I7963	I13798	I14747	I18835
I2382	I15823	I8365	I15941	I7964	I15041	I12878	I18220
I2380	I16108	I6695	I1382	I15646	I15957	I19863	I18226
I0409	I3068	I6696	I7281	I16110	I13799	I13729	I12906
I7951	I3033	I1271	WEHR_1192SkA	I2609	I13783	I16453	I2695
I7952	I16435	I1276	I4946	UNTA58_153	I17611	I14551	I16422
I15648	I0559	I1280	I3590	AITI_36	I13786	I19722	I5506
I15819	I6747	I1282	SUC004	WEHR_1586	I16182	I12772	I2694
I15818	I6755	I5838	I7211	I0115	I13794	I16439	I20752
I15821	I1593	I1284	I1391	MX252	I15959	I16440	I19210
I1896	I7601	I1300	I0059	I2601	I15960	I16441	I21402
I0412	I6759	I1303	I4945	I14553	I15956	I16442	I19359
I1887	I13891	I1314	I16169	I6610	I15958	I16380	I19357
I1894	I6746	I6617	I3600	I20063	I17612	I16388	I20827
I1506	I6761	I6628	I3601	I7639	I13795	I12771	I19356
I0413	I3039	I6629	I3588	MX277	I16538	I16591	I19361
CHA001_merged	I3040	I6630	I3589	I2421	I16113	I15952	I20828
CHA002	I2635	TU876(SX10)	I3594	WEHR_1192SkB	I17671	I18259	I21401
I26738	I7644	I16247	ISC001	I2610	I13789	I15046	I19358
I0410	I13893	MX191	I5755	I3494	I13781	I15954	I19362
I1498	I3135	I0826	I5757	I12776	I13791	I15044	I20813
I5068	I5366	I1392	I5759	AITI_72	I17311	I17614	I21399
I1499	I15826	MX198	I16448	JK2723	I13793	I14100	I20815
CHA003	I6757	I0823	I4885	I12855	I13796	I11699	I20817
I2199	I12313	I0258	SUA005	I12809	I13792	I11701	I21931
I3535	I0560	I0260	HUGO_168	I2463	I13788	I11708	I19363
I1500	I3134	I0261	I4896	AITI_50	I20515	I3757	I19360
I1505	I4308	I0262	I4229	I4067	I16089	I21305	I20812
LBR005	I12317	I0263	I5514	I18606	I16549	I16599	I21400
I1882	I3133	I1553	I6480	I15033	I3642	I11148	I21403
I4196	I3041	I0457	I6468	I4076	I25523	I11153	I20811
I2743	I5079	RA54	I4139	TU911(SX23)	I5383	I16597	I20816
I2744	I7272	MX196	I6775	I2602	I16469	I15049	I18839
I2745	I4949	I5374	I6776	AITI_66	I12936	I17613	I18527
I4186	I7598	I0103	I6778	AITI_92	I16459	I19872	I18528
I4187	I18428	I2630	SUC005	AITI_43	I16467	I14808	I18840
I3536	I18427	I0550	PJU002	I19220	I12608	I5696	I18526
I2378	I2605	I13467	I12786	I4884	I13711	I13732	I18529
I4188	I3136	ALT_1	I4889	MX279	I10364	I16611	I18531

I2355	I2988	MX298	I4891	I5377	I13712	I21180	I18110
I2376	I2636	I8131	I2453	I4892	I13713	I14549	I18988
I2375	I18426	I7953	EHU001	AITI_2	I24345	I13685	I12790
I4199	I7594	I6467	I6774	I16792	I24344	I14378	I12793
I1496	I2660	I0257	I0171	I5441	I7688	I21293	I21271
OBN006	I8568	I2932	I2568	I21306	I7687	I13717	I21272
FUC003	I2650	I1976	I4895	AITI_78	I12614	I2983	I21274
I1904	I2637	ALT_2	I16424	I4420	I12611	I14986	I21275
I0176	I8567	I1539	HUGO_190	AITI_119	I12612	I15045	I21276
I2377	I3137	I16164	I11159	AITI_40	I12613	I17145	I21277
LBR002	I6753	I4144	I6476	AITI_77B	I14742	I14987	I19854
I2379	I3023	I0462	I5042	MX257	I14377	I17669	I19855
I5069	TGM009	I1277	I5037	I16791	I1504	I14985	I12792
I5208	I3214	I0108	I6613	JK2739	I20525	I14984	I0527
I7950	I2369	MX188	I6622	I6618	I20526	I20518	I12926
I15650	I7554	HUGO_169Sk1	I6623	I7630	I14481	I17146	I19037
I1891	I0519	I16438	I6543	AITI_62A	I17310	I14988	I19040
I1893	I0518	I5748	I6609	AITI_65adult	I25504	I14858	I19043
OBN005	I0520	I7207	I6612	I4071	I25505	I13683	I11993
I1890	I2752	I3528	RA64	I2981	I3130	I20519	I11994
I4185	I2755	I1389	I0117	OTTM_154	I2861	I20522	I19655
LBR004	I2785	I0104	I2443	I4070	I14864	I20504	I14809
I4184	I2980	AMP_1	I4145	I1774	I14862	I17315	I16619
LBR001	I2763	I18968	HUGO_167	I8144	I2469	I20523	I20624
I1972	I2371	MX197	I4886	AITI_95	I2860	I13780	I20622
OBN002	I5428	I0459	MX193	I6680	I14865	I16269	I15039
OBN010	MX212	I18967	I2596	I18945	I20062	I16273	I15953
I0449	RA42	UNTA58_68Sk2	I2597	AITI_120	I14861	I17316	I18181
I2387	I0800	SUC006	I2566	I0210	I2859	I16270	I18183
I2358	I16183	I0825	I5665	I0206	I3315	I17317	I18182
I2746	MX150	TU904(SX17)	JK2716	I0207	I14358	I20521	I17266
GRG027	I0551	SEC004	I20997	I0209	I14379	I20509	I19657
I1900	I0802	I6605	I3484	I1312	I12624	I17320	I11152
LBR003	I2651	I7278	I2604	I1310	I5697	I17321	I12907
I4305	MX182	I4329	I5382	VAD004	I14745	I17322	I17262
GRG028	I0172	I5379	I3874	VAD002	I23911	I17313	I20987
TU916(SX30)	FIL004	I1390	I3256	VAD003	VIL011	I17323	I20983
I4304	I3019	SEC006	I5471	I3997	I24883	I17324	I20984
I4167	I3085	I14677	MX189	VAD001	I23996	I17314	I20982
TU919(SX33)	I5076	I14200	I2457	I5516	VIL010	I20513	I20985
I4303	I1594	I2467	I2452	I2464	I23995	I17327	I20986
GRG057	I2754	TU908(SX21)	MX288	I4562	I11683	I16271	I25524
OBN001	I2978	I0118	I10345	I6470	I18241	I16268	I3326
TU915(SX29)	I2934	I7208	I10343	AITI_98	I18213	I16272	I3327
GRG018	I19286	I2575	I10342	I3487	I18245	I17325	I20630
I4168	I1838	TU914(SX26)	I10344	OTTM_151ind1	I18211	I20507	I20634
I26741	MX213	SUC007	I10347	VAD005	I18216	I20520	I20621
GRG049	I2370	I4245	I10348	TU907(SX20)	I18246	I20503	I20631

I0634	RA43	I5367	I5750	I3488	I18239	I20510	I20632
I1906	I2935	I4131	I3485	I3486	I3313	I20511	I12411
I2352	TGM008	I0111	I8048	I7570	I5692	I19916	I13755
I1899	I2979	I4247	I6777	I1775	MSR002	I13620	I13756
I1901	I5118	I4947	I2445	I4559	I5693	I13623	I13757
OBN011	I5116	I3529	I1767	I4560	I24638	I19917	I14099
OBN003	I8566	I7040	I2454	I4561	I24639	I19918	I5504
OBN008	I6764	I7044	I7199	I1836	I26742	I13621	I5505
OBN004	I6766	I4178	MX251	I7573	I23904	I13720	I13758
FLR004	I5119	I3243	I13027	I4331	I15071	I4998	I13760
I1131	I2753	I3239	I4888	OTTM_141A	I16088	I13615	I12413
I14190	I2606	I3238	I23569	I4332	I16087	I19873	I14101
GRG008	I11599	I2365	I5373	I2471	I16086	I19874	I14103
GRG015	I11601	I0106	I4074	I7569	I14983	I19907	I14104
GRG021	I11248	I15942	I7635	I1840	I14980	I19908	I14107
GRG023	I11249	I0049	I7198	I1977	I14982	I19910	I13753
GRG025	I11300	I2612	I1502	I8045	I16326	I19911	I13754
GRG035	I11301	TU918(SX32)	I11158	I3490	I16327	I20586	I11034
GRG041	I7602	HUGO_180Sk1	I8582	I3491	I16329	I20589	I12414
GRG047	I7603	I0460	I14675	I3492	I17607	I21178	I12415
GRG056	I7604	I4124	SUC001	OTTM_156	VIL006	I19211	I5508
GRG003	I7605	I2786	I2567	I16161	I13682	I21181	I14105
GRG019	I7606	I7209	I7249	I2639	I12171	I21182	I14108
GRG022	I1497	I4143	I7043	I26830	I5691	I19209	I13751
GRG043	I15034	I0839	I0804	I19858	I16618	I19856	I14102
GRG050	I15035	I2787	OBKR_80	I2472	I16092	I19653	I13759
GRG052	I3138	I4136	I12935	I8136	I12903	I19654	I16596
FLR014	MX211	I5666	I2598	I16403	I23974	I11146	I13681
FLR001	RA45	I4141	POST_47	I13714	I24882	I16405	I20989
GRG016	Aes4	I4130	I7638	I19857	I24879	I2982	I15047
I0166	I3272	I7202	I3255	I19915	I12641	ORC002	I21309
OBN009	I2631	I7200	I2618	I24342	I12783	I14802	I21313
OBN007	I3277	I7201	I4073	I19859	I19867	I19652	I2696
FLR003	MX210	I7203	I16412	I26828	I19861	I13731	I20584
I4894	MX184	I7195	I3875	I26829	I14743	I19042	I13616
I1495	MX203	I7196	I3743	I26831	I5698	I10366	I25509
FLR013	MX204	I4890	I4068	I26832	I27382	I20587	I25510
TU877(SX11)	MX209	I1381	I0116	I7568	I16617	I13727	I25512
I4893	TU910(SX22)	I5520	I2569	I19860	I13688	I14807	I25516
FLR010	Aes16	I5521	I0803	I3082	I12610	I11147	I25518
TU875(SX9)	Aes1	UNTA58_68Sk1	I14543	I5440	I19868	I15043	I25519
FLR007	I2611	I5524	I2461	I11972	I16163	I15040	I25522
FLR005	I16429	I5523	POST_2	I7572	I11719	I18492	I25517
LON003_LON004	I16425	I5525	I2600	I2573	I5690	I18489	I8205
I2394	I1975	I5527	I4069	I16184	I19862	I18491	I8203
PRI001	I3432	I5529	MX256	I16476	I19287	I18493	I3320
I2353	Aes3	I4134	SUC003	I7579	MX265	I18488	I22065
I2354	I5358	I5531	POST_99	I16170	I8209	I18490	I16613

I2395	I4565	I6481	I13025	I2458	I12774	I7632	I11143
PRI005	I14326	I6482	I13026	I26893	I12643	I19045	I14837
PRI006	Aes5	I5836	I16782	I26774	I25507	I12412	I13726
NOE002	I1843	I13028	I4075	I26773	I25525	I22056	I20628
I7550	Aes13	I5513	I16395	I16112	I12640	I22055	I13752
I8134	I10277	I2417	I16396	I16099	I4556	I13730	I14353
SID005	I10278	I5512	I1770	I16100	I12560	I16595	I16503
I16437	I10280	I4951	I2455	I16111	I12561	I4996	I20626
NOE001	I10283	I4950	I6604	I17670	I17260	I14380	I16620
GRG032	I10284	I2450	POST_85	I2655	I20585	I19914	I21307
I1909	I10285	I7212	I2462	I7640	I26628	I3083	I11991
I7042	I10286	I14678	I2447	I7571	I6769	I20583	I21310
I16427	I11303	MX259	I6679	I3493	I5689	I14804	I17263
I4189	I11305	I2741	I2413	I12082	I13689	I19207	I16416
I1908	I11306	I0840	OBKR_50	I5695	I8215	I20620	I22052
I16444	Aes14	I0461	OBKR_93	I2654	I18227	I14860	I8206
I2783	I1981	I12900	MX280	I11973	I11717	I16592	I20766
I18691	Aes2	I0453	I3132	I2574	I11721	I19656	I20767
FLR002	Aes17	I2416	POST_16	JK2714	I11722	I18530	I21312
I15946	I2933	I18970	OBKR_6	I2470	I12098	I12778	I11992
I7547	I5429	I2565	OBKR_66	I16616	I12099	I14806	I21314
I7549	RA58	I2418	POST_28	I7574	I12103	I11156	I20625
I7647	RA62	MX199	MX258	I19913	I12105	I19044	I16430
I7645	Aes8	I7213	UNTA85_1412	I17019	I12106	I19046	I16601
I15945	I3269	I7045	OBKR_82	I7627	I12107	I14800	I14327
SUC008	I3270	I2459	OBKR_2	I12081	I12110	I21179	I16609
I16436	I3271	I5385	OBKR_67	I13710	I14465	I16593	I21311
I2657	I1842	I27380	I0164	I12208	I14467	I11150	I22060
I6748	I5387	I1549	UNTA58_149	I12209	I14468	I11151	I16499
ELT006	Aes9	I1546	SUA007	I16479	I5287	I13728	I27379
I16168	I2977	I1536	OBKR_9A	I10552	I5288	I11997	I11142
I7643	Aes20	I1538	AITI_70	I7626	I12097	I20627	I5503
I2658	Aes24	I1540	I15824	ORC004	I12102	I14859	I2693
I0807	Aes18	I1542	MX270	I2653	I12104	I19909	I16505
I6760	Aes10	I1544	MX254	I13716	I14464	I17015	I21308
ELT002	RA44	I1532	I20750	I18719	I27383	I17014	I16495
I16166	I1845	I0112	I7689	I20751	I13690	I3758	I5502
I15029	I1978	I7286	I7691	I20749	MSR003	I8208	I11716
I15030	I1846	I2364	I7692	I11629	I12905	I3323	I12785
I15031	Aes21	I15028	I23209	I20058	I3322	I14866	I12791
I15032	I2473	I0806	I23210	I2448	I16498	I17016	I19870
I0405	I7642	I5376	I23211	I7577	I27381	I16475	I17017
I0406	RA57	I2446	I23206	ORC003	I26630	I16514	I19989
I0407	RA61	UNTA85_1343	I23208	ORC007	I11154	I16410	I19991
I6762	I4175	HUGO_171	I23212	ORC001	I23978	I3014	I16450
I16463	I2629	I7269	I23207	I16454	I11995	I12770	I16455
I3035	Aes6	I7270	I23205	ORC005	I16602	I12775	I16386
I0408	UNTA121_FK61	I7275	I16400	SUA006	I22937	I12779	I16457

I2788	I11604	I7276	OBKR_117	ORC006	I12642	I20623	I16458
I13892	I11605	I7250	I5515	I10554	I11149	I20988	I16456
I1565	Aes7	I7279	OBKR_84	I13618	I14381	I17264	I16387
I16165	Aes22	I7280	I2460	JK2720	I17259	I17267	I16390
I3005	I0455	I7251	POST_6	I20057	I2692	I14863	I19587
I2789	Aes23	I16090	OBKR_76	I10553	I12879	I20588	I14351
I6751	I3499	I7205	I3756	I7575	I22940	I17261	I14352
I6750	UNTA89_FK231	I7282	I15825	I12083	I22938	I14803	I11712
I2633	I8569	I7287	I14193	I20735	I8211	I13680	I11713
I2659	I16491	I7288	I14185	I20736	I11033	I14347	I11715
I21390	I2792	I7290	I14585	I20740	I16612	I3324	I14106
I7600	SID006	I7214	I14191	I3741	I17258	I3759	I19869
I13889	I1281	I5519	I14186	I7580	VIL007	I13687	I21315
I12312	I6601	I6591	I14189	I13617	I14857	I19912	I6771
I7646	I11592	I6590	I14192	I7578	I13684	I14348	I3567
I2691	I11614	I5658	I11160	ORC009	VIL009	I15951	I19990
I21385	I8148	I5659	I14188	I11971	I12877	I15042	TU905(SX18)
I13897	I8153	I5834	I15642	I16488	I8344	I17616	I17750
I13899	I8158	I6624	I7960	I7576	I16600	I17143	I3566
I13890	I8156	I5833	I13470	I7628	I12787	I17139	I19588
I5359	I8149	I5014	I15643	I16615	I15950	I20990	I16471
I5371	I8154	I5023	I7958	ORC008	I15048	I20582	I11145
I19987	I14097	I2799	I14359	I2699	I12927	I16413	I10866
I21303	I0525	I5365	I22062	I3568	I12931	I16504	I14552
I26629	I14096	I16418	I18599	I2824	I12932	I27385	I27384
I14360	I22064	I11631	I21302	I14550	I5474	I22057	I11710
I19047	I11144	I20615					

Supplementary table 3 | Phenome-wide associations with FUT2 truncation allele rs601338-A. Data from Finngen freeze 11. Phenome-wide was defined as the Bonferroni-corrected threshold of p<0.05/2,445 phenotypes.

**Phenotype**	**Effect size**	**P value**	**N(cases)**	**N(controls)**
Viral and other specified intestinal infections	-0.146007	8.8e-22	9801	403431
Intestinal infectious diseases	-0.0577324	1.1e-16	50302	403431
Chronic sinusitis	0.0755342	1.4e-12	19856	337881
Diarrhoea and gastroenteritis of presumed infectious origin	-0.0532175	4.8e-11	35960	403431
Cholelithiasis	0.0485001	1.3e-10	44582	397583
Type1 diabetes, definitions combined	0.141591	2.9e-10	4526	369007
Type1 diabetes, definitions combined, early onset	0.172778	4.1e-10	2947	450786
Autoimmune diseases	0.0315999	6.1e-10	115447	338286
Acute laryngitis and tracheitis	0.121135	1.8e-9	5370	366465
Cholecystectomy	0.0492359	7.4e-9	32536	421197
Benign neoplasm: Colon	0.0581571	3.9e-8	20424	433309
Statin medication	0.0290167	6.9e-8	155745	297988
Peritonsillar abscess	0.0821586	8.1e-8	9339	337881
Other diseases of upper respiratory tract	0.02644	1.6e-7	115852	337881
Benign neoplasm of colon, rectum, anus and anal canal	0.0492006	3.3e-7	25190	428543
Autoimmune diseases excluding thyroid diseases	0.0323869	3.7e-7	63967	337247
Autoimmune diseases excluding thyroid diseases, strict definition	0.0316975	8.8e-7	62294	331969
Benign neoplasm: Caecum	0.123711	1.9e-6	3121	450612
Diseases of middle ear and mastoid	-0.0406161	4.2e-6	31326	422407
Chronic rhinitis, nasopharyngitis and pharyngitis	0.0584506	5.1e-6	13519	337881
Hypothyroidism, strict autoimmune	0.0332728	7.3e-6	49055	329775
